# Quantitative Assessment of rPM6 for Fluorine- and Chlorine-Containing Metal Complexes: Comparison with Experimental, First-Principles, and Other Semiempirical Results

**DOI:** 10.3390/molecules23123332

**Published:** 2018-12-15

**Authors:** Toru Saito, Manami Fujiwara, Yu Takano

**Affiliations:** Graduate School of Information Sciences, Hiroshima City University, 3-4-1 Ozuka-Higashi, Asa-Minami-Ku, Hiroshima 731-3194, Japan; fujiwara@bio.info.hiroshima-cu.ac.jp (M.F.); ytakano@hiroshima-cu.ac.jp (Y.T.)

**Keywords:** semiempirical method, magnetic exchange coupling, oxidation reaction

## Abstract

We report a reparameterization of PM6 parameters for fluorine and chlorine using our training set containing transition metal complexes. Spin unrestricted calculations with the resulting rPM6 (UrPM6) were examined quantitatively using two test sets: (i) the description of magnetic interactions in 25 dinuclear metal complexes and (ii) the prediction of barrier heights and reaction energies for epoxidation and fluorination reactions catalyzed by high-valent manganese-oxo species. The conventional UPM6 and UPM7 methods were also evaluated for comparison on the basis of either experimental or computational (the UB3LYP/SVP level) outcomes. The merits of UrPM6 are highlighted by both the test sets. As regards magnetic exchange coupling constants, the UrPM6 method had the smallest mean absolute errors from the experimental data (19 cm^−1^), followed by UPM7 (119 cm^−1^) and UPM6 (373 cm^−1^). For the epoxidation and fluorination reactions, all of the transition state searches were successful using UrPM6, while the success rates obtained by UPM6 and UPM7 were only 50%. The UrPM6-optimized stationary points also agreed well with the reference UB3LYP-optimized geometries. The accuracy for estimating reaction energies was also greatly remedied.

## 1. Introduction

**Scheme 1:**
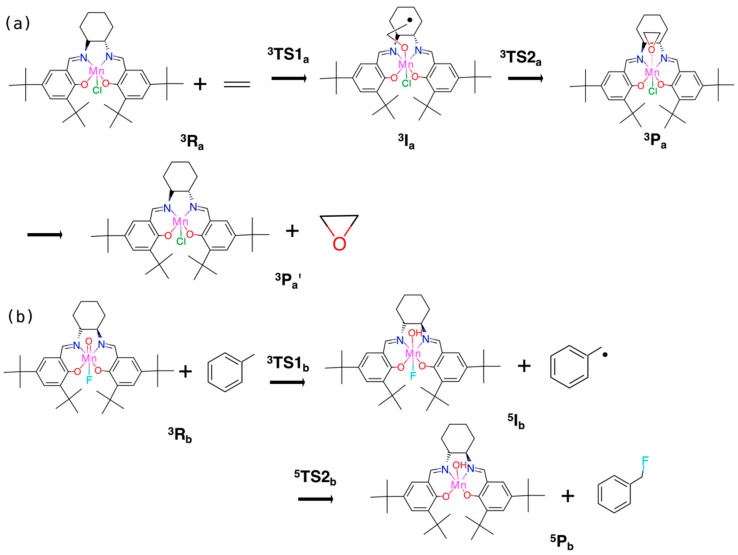
(**a**) Ethylene epoxidation catalyzed by ^3^[Cl–Mn^V^=O(salen)] and (**b**) toluene fluorination catalyzed by ^3^[F–Mn^V^=O(salen)]. The superscripts 3 and 5 on the left side denote triplet and quintet spin states, respectively.

In oxidation reactions, mononuclear metal-oxo species such as Fe(IV)=O and Mn(IV)=O are considered to be key intermediates that activate inert C–H and C=C bonds to generate alcohols and epoxides [1]. Halogen atoms may be involved as axial ligands in an enzyme active site, and can control the catalytic reactivity. Biomimetic studies sometimes introduce halogen atoms for the purpose of enhancing their target reactions [2,3,4,5]. For instance, chiral Mn^III^-salen complexes achieve enantioselective epoxidation of olefins, so-called the Jacobsen-Katsuki epoxidation (see Scheme 1a) [2,3]. This type of epoxidation reaction is expected to start with the attack of a Mn^V^=O on one of the carbon atoms of the C=C bond. The chloride ion is crucial to keep conversion rates, especially for an analogue without substituents [4]. 

Among the halogens, fluorine can play an important role in drug design and development [6,7]. The notable physical properties of fluorine are its high electronegativity and small van der Waals radius, thus, fluorine-containing molecules affect the metabolism of drugs and show high bioavailability. The ^18^F isotope-labelled molecules, and above all, 2-[^18^F]-fluoro-2-deoxy-d-glucose, are widely used as positron emission tomography tracers [6]. Since the number of fluorine-containing natural products are limited, many strategies for introducing fluorine into a molecule have been proposed including fluorination and trifluoromethylation reactions [8,9,10]. Groves and co-workers developed a late-stage process for the ^18^F fluorination into the benzylic position of a molecule [10]. They revealed that a Mn(salen) complex with *p*–toluenesulfonate significantly outperforms that with chloride, affording the highest radiochemical conversions. The reactive species of this benzylic C–H fluorination process may be mediated by [F–Mn^V^=O(salen)], responsible for hydrogen abstraction as depicted in Scheme 1b. 

Polynuclear complexes may catalyze challenging oxidation reactions with high selectivity [11,12,13]. However, even for dinuclear systems, less information is available on the electronic and geometric structures of reactive intermediates compared to mononuclear complexes. Spectroscopic studies of magnetic exchanges between spin centers are capable of contributing to the characterization of an active species [14,15]. In addition to catalytic activities, magnetic properties of polynuclear complexes have also been studied with the aim of developing promising candidates for quantum computing and high-density magnetic storage devices [16,17]. To understand fundamentally the mechanism of magnetic coupling, studies on dinuclear complexes with fluoride and chloride bridges have been carried out as well as those with oxo and hydroxo bridges [18,19,20,21,22,23,24,25,26,27,28,29,30,31,32,33,34,35,36,37,38]. 

On the theoretical side, the first-principles spin unrestricted density functional theory (UDFT) calculations may help determine reaction pathways by locating transition state (TS) structures and by computing activation barriers and reaction energies [39]. The computational prediction of magnetic exchange coupling constant (*J*) values is also one of the important issues, because the sign and magnitude of the magnetic property is sensitive to a change in electronic structures and local geometries of metal centers [40,41,42,43]. Unlike thermochemical properties, few benchmarks have been established using semiempirical quantum mechanical (SQM) methods, not to mention transition-metal-containing complexes [44,45,46,47]. The reason is simple: there is no SQM method that has been designed for strongly correlated electron systems. 

**Figure 1:**
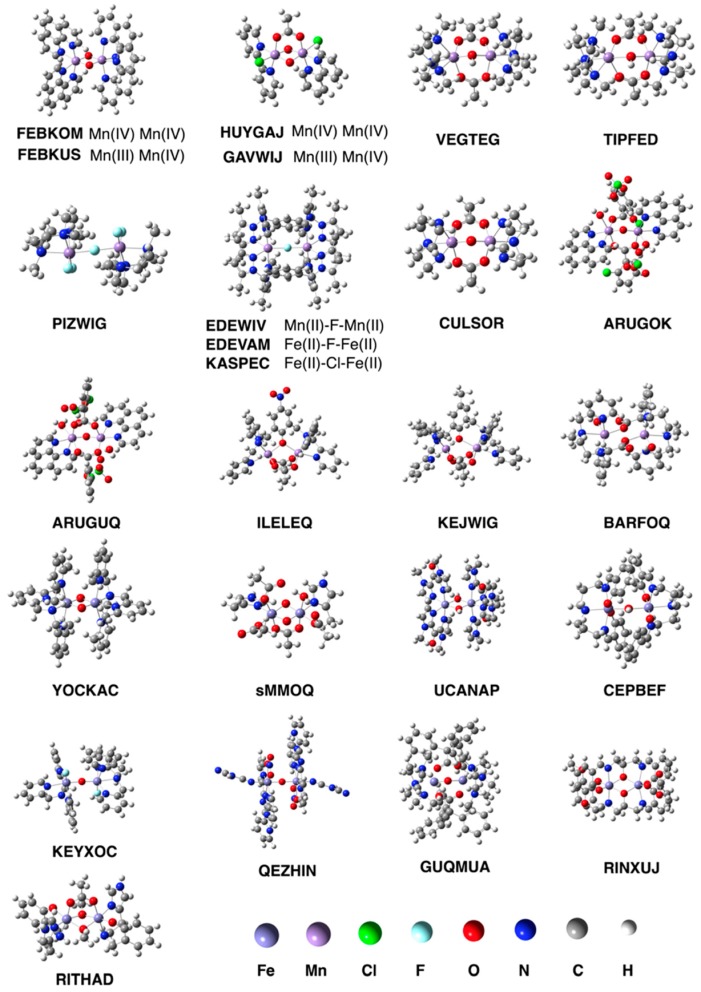
Twenty-five dinuclear manganese and iron complexes (for details, see also Tables S1 and S2).

Very recently, we proposed the rPM6 method [48,49,50,51,52], which is a reparameterization variant of PM6 [53], and tested the spin unrestricted PM6 (UrPM6) against organic diradicals and iron– and manganese–catalyzed C–H and C=C bond activation reactions. It turned out that UrPM6 outperforms the original UPM6 and spin unrestricted density functional-based tight binding (UDFTB) methods for calculating *J* values for organic diradicals. The examination on the oxidation reactions also demonstrated its improved performance for TS searches. In the present work, the reparameterization and subsequent evaluations of the parameter sets for fluorine and chlorine are reported. We check whether our UrPM6 can be applied to the quantitative prediction of magnetic interactions, reaction energies, and barrier heights on the basis of a test set. One test set contained 25 dinuclear complexes associated with magnetic coupling (see text below and Figure 1). The other set was composed of the two oxidation reactions as already noted above (see Scheme 1). 

## 2. Computational Details

### 2.1. rPM6 Parameters for F and Cl

The reparameterization of PM6 involves the optimizations of electronic parameters and parameters for the core-core interaction. The electronic parameters for fluorine are composed of the one-electron one-center integrals ($U_{ss}\;\mathrm{and}\;U_{pp}$), the resonance integrals ($\beta_{s}\;\mathrm{and}\;\beta_{p}$), and the Slater orbital exponents ($\zeta_{s}\;\mathrm{and}\;\zeta_{p}$). Due to hypervalent compounds, chlorine includes the additional three parameters for d-orbitals ($U_{dd},\;\;\beta_{d},\;\mathrm{and}\;\;\zeta_{d})$ [54]. The optimization was not performed for the other electronic parameters including one-center two-electron integrals, in analogy with Pairwise Distance Directed Gaussian (PDDG)/PM3 [55]. As such, the six and nine electronic parameters were optimized for fluorine and chlorine, respectively. The core-core interaction is written using the charge of the nucleus ($Z_{i}$), the two-electron two-center integral ($\langle s_{i}s_{i}|s_{j}s_{j}\rangle$), the distance between atoms *i* and *j* ($R_{ij}$), and the optimizable Voityuk’s diatomic core-core parameters ($\alpha_{ij}$ and $x_{ij}$) [56]:

(1) $$E_{n}\left(i,j\right)=Z_{i}Z_{j}\langle s_{i}s_{i}|s_{j}s_{j}\rangle \left(1+x_{ij}e^{-\alpha_{ij}\left(R_{ij}+0.0003R_{ij}^{6}\right)}\right)$$

Using this equation meant that the two parameters corresponding to each atom pair, such as F–O and Cl–Mn, were optimized. Fluorine and chlorine both had 12 optimizable core-core parameters, resulting in the 18 and 21 parameters in total for fluorine and chlorine, respectively (for details, see Table 1). During the optimization, the parameters for H, C, N, O, Mn, and Fe remained frozen at the values determined in our previous studies [48,49,52].

The parameters for fluorine were first refined on the basis of the training set **1**, followed by those for chlorine using training set **2**. Details of the training sets **1** and **2** are collected in Table S1 and Figure S1 in the Supplementary Materials, and are summarized as follows:

**1.**  Geometric parameters of four open-shell and 11 closed-shell species [9,57,58] with 72 reference data points. Nine subsets for general chemical problems taken from the GMTKN30 database with 52 reference data points, which covered ionization potential (G21IP), electron affinity (G21EA), atomization energies (W4-08), decomposition energies (MB08-165), proton affinities (PA), barrier heights and reaction energies (BH76, BH76RC, and G2RC), and radical stabilization energies (RSE43) [59,60,61]. The total number of reference data points (*N*) is 124.
**2.**  Geometric parameters of three open-shell and 15 closed-shell species [56,62,63,64] with 86 reference data points. Nine subsets for general chemical problems taken from the GMTKN30 database with 50 reference data, which covered ionization potential (G21IP), electron affinity (G21EA), atomization energies (W4-08), decomposition energies (MB08-165), barrier heights and reaction energies (BH76, BH76RC, and G2RC), self-interaction error related problems (SIE11), and isomerization energies (ISOL22) [59,60,61]. The number of reference data points (*N*) totaled 136.

For each training set, reference data for geometric variables were obtained at the UB3LYP/SVP level of theory [65,66,67,68] using the Gaussian 09 program package [69]. Then, a response function *S*, which represents a sum of the square of the difference between calculated values ($Q_{\mathrm{calc}}\left(i\right)$) and reference data ($Q_{\mathrm{ref}}\left(i\right)$), was minimized to find the best set of parameters **P**:

(2) $$S=\sum_{i}^{N}{\left(Q_{\mathrm{calc}}\left(i\right)-Q_{\mathrm{ref}}\left(i\right)\right)}^{2}.$$

### 2.2. Evaluation of rPM6 on Calculating Magnetic Interactions, Reaction Energies, and Barrier Heights

Upon obtaining the optimized parameter sets, we evaluated the applicability of UrPM6 to the quantitative prediction of magnetic interactions, reaction energies, and barrier heights. The original UPM6 and UPM7 methods were also tested for comparison [70]. As aforementioned, our test set specifically consisted of (i) dinuclear 14 dimanganese (from FEBKOM to BARFOQ) and 11 diiron (from YOCKAC to RITHAD) complexes corresponding to the Cambridge Structural Database reference codes as depicted in Figure 1 [18,19,20,21,22,23,24,25,26,27,28,29,30,31,32,33,34,35,36,37,38], and (ii) an ethylene epoxidation reaction catalyzed by ^3^[Cl–Mn^V^ = O(salen)] and a toluene fluorination reaction catalyzed by ^3^[F–Mn^V^=O(salen)] [2,10,71]. 

Note that the superscript 3 on the left side denotes a triplet state. Concerning the test set (i), geometry optimizations were carried out on the low-spin (LS) states, except those for CULSOR and ARUGOK, which exhibit ferromagnetic interactions, that were performed on the high-spin (HS) states. For the ARUGOK and ARUGUQ systems, two ClO_4_^=^ counter anions were included in the models because they form hydrogen bonds with the water molecules coordinated to Mn. To calculate *J* values using an approximate spin projection formula in Equation (3) [72,73,74,75], the single point energy calculations were performed at the optimized geometries on the corresponding HS states of 23 metal complexes and LS states of CULSOR and ARUGOK.

(3) $$J=\frac{E^{\mathrm{LS}}-E^{\mathrm{HS}}}{\langle {\hat{S}}^{2}\rangle {}^{\mathrm{HS}}-\langle {\hat{S}}^{2}\rangle {}^{\mathrm{LS}}}$$

For the test set (ii), geometry optimizations were performed to locate local minima and TS structures. We assessed whether the results of the SQM methods agree with those of the density functional theory (DFT) in the optimized geometries of the stationary points, reaction energies, and barrier heights. In the present study, the UB3LYP/SVP model was chosen as the reference. All the quantum chemical calculations were done with the Gaussian 09 (UPM6, UrPM6, and UB3LYP/SVP) [69] and Gaussian 16 (UPM7) program packages [76], while reparameterizations were carried out by our own code [48]. The geometry optimizations were performed using the default convergence criteria.

## 3. Results and Discussion

Two sets of optimized parameters, for rPM6 and those for the original PM6, are presented in Table 1. 

This section continues with the calculated results of magnetic interactions, followed by those of the ethylene epoxidation reaction and toluene fluorination reaction.

### 3.1. Test Set (i): Magnetic Interactions

Table 2 summarizes the computed and experimental *J* values for 25 dinuclear metal complexes used as the test set (i). The statistical evaluation based on mean absolute errors (MAEs) and mean absolute relative errors (MAREs) is also presented. Note that the results of ARUGUQ and BARFOR are excluded from the statistical analysis because geometry optimizations for these complexes failed using UPM6 and/or UPM7. The first four systems, namely FEBKOM, FEBKUS, HUYGAJ, and GAVWIJ, contain a [Mn_2_(μ–O_2_)] unit responsible for an antiferromagnetic interaction with a negative *J* value. The pairs FEBKOM and FEBKUS as well as HUYGAJ and GAVWIV differ in their oxidation states of each of the Mn ions. 

The experimental observations indicate the mixed-valence Mn^III, IV^ complexes exhibit a stronger antiferromagnetic coupling, and the existence of Cl atoms may make the trend more prominent (−45 vs. −114 cm^−1^). The *J* values calculated by both the conventional UPM6 and UPM7 methods are significantly different from the experimental values. The UPM6 method gave a positive *J* value for HUYGAJ, which is qualitatively incorrect. The latest UPM7 method can qualitatively reproduce the antiferromagnetic couplings, but the calculated data considerably underestimated their strength. Our UrPM6 method is obviously superior to these conventional ones in that the quantitative agreement with the experimental measurements was also obtained, albeit with a slight overshoot of the *J* value for HUYGAJ. 

Unlike the bis(μ–oxo) species, the magnetic interactions in μ–*X*–bridged (*X* = OH, O, F) systems are weak regardless of the oxidation states of Mn ions, as was found, for example, in TIPFED and ARUGOK. All SQM methods examined here performed relatively well for the VEGTEG and TIPFED complexes with the [Mn–OH–Mn] core, whereas substantial deviations were given in the Mn–F–Mn systems with the UPM6 and UPM7 methods. The performance of the original UPM6 was patchy. It worked well for PIZWIG with a small absolute error of 1 cm^−1^, but failed totally to describe the interaction in EDEWIV. The UPM7 method tended to overestimate the antiferromagnetic interactions significantly for both the PIZWIG and EDEWIV. We found that a possible origin of these errors was a large deviation in the optimized structures. As can be seen in Figure S2, the bridging F atom in the UPM6-optimized EDEWIV complex resulted in binding to only one of the Mn ions. The optimized distances between Mn and F were 1.86 and 3.76 Å, which was far from those in the X-ray crystallographic data (2.07 and 2.07 Å). On the other hand, UPM7 provided a short Mn–F distance (1.78 and 1.72 Å), leading to a stronger superexchange interaction with a *J* value of −121 cm^−1^. The overestimation of the magnitude was also observed for PIZWIG with the [Mn^III^–F–Mn^III^] core (−470 cm^−1^). UrPM6 succeeded in yielding reasonable estimates of *J* values for Mn–F–Mn systems as opposed to UPM6 and UPM7, because of its much better reproducibility of the structures. 

The superior performance of UrPM6 was also found in the Mn–O–Mn complexes, where CULSOR and ARUGOK showed weak ferromagnetic behavior. The experimentally observed *J* value for ARUGUQ (−13 cm^−1^) was closer to the result of UPM6 (−8 cm^−1^) than to that of UrPM6 (−35 cm^−1^), but the agreement must be fortuitous. In fact, unlike UrPM6, the UPM6 calculation was unable to reproduce ferromagnetic coupling for its spin isomer, i.e., ARUGOK. The ILELEQ and KEJWIG complexes are classified as a phenolate-bridged dimanganese complexes, while the two acetates bridge the Mn ions in BARFOQ. The UrPM6 method outperformed UPM6 and UPM7 as far as ILELEQ and BARFOQ are concerned. All the SQM computations predicted that the KEJWIC complex may exhibit a weak ferromagnetic coupling, which is incorrect. Such an inaccuracy is not surprising since estimating *J* values for complexes with a small energy splitting is challenging even for UDFT [40,41,42,43].

Now let us look at the performance of the three methods for diiron complexes. The presence of a high-valent [Fe^IV^_2_(μ–O_2_)] core in sMMOQ is known to show a relatively strong antiferromagnetic coupling (*J* < −30 cm^−1^), but no specific value was determined. Here, we took a DFT-calculated value of *J* = −224 cm^−1^ as the reference provided in the literature [77]. It is obvious that the magnitude of the *J* values obtained with UPM6 were much larger than those obtained with experiments by at most two digits, excluding the case of KASPEC. Our recent study revealed that the UPM6 optimization made a Fe···Fe separation in sMMOQ too short [52], and the same is also true for other diiron complexes. As such, the poor reproducibility in the geometries of the diiron centers is responsible for the overwhelmingly large deviations of *J* values. The calculated results of UrPM6 and UPM7 were much closer to the experimental values, presumably due to the improved description of Fe–Fe repulsion in a LS state. Nevertheless, the weak antiferromagnetic interactions in YOCKAC as well as EDEVAM and KASPEC were not treated properly by UPM7. The ligands of the latter two complexes are the same as that of EDEWIV (see Figure 1). This clearly demonstrates that UPM7 is not suitable for fluoride- and chloride-bridged complexes, though the average differences in the Fe–F and Fe–Cl distances between the computation and the experiment (0.06 and 0.07 Å) were much smaller than the Mn–F counterparts in EDEWIV (0.32 Å). By contrast, such problems were not seen when using UrPM6. Its performance for all of the diiron complexes was stable and not susceptible to the type of bridging species.

Overall, on the basis of the statistical errors (MAEs and MAREs), our UrPM6 method performed the best, followed by UPM7 and UPM6. The subsets including diiron complexes caused considerable increases of these errors for UPM6, while fluoride- and chloride-bridged systems, namely PIZWIG, EDEWIV, EDEVAM, and KASPEC, deteriorated the reliability of UPM7. If these subsets are disregarded from the statistical evaluation, the MAEs/MUREs are reduced to 67.0/13.0 and 96.0/3.3 for UPM6 and UPM7, respectively. The large MAEs obtained with UPM7 are attributed to its poor performance for YOCKAC. The UrPM6 method was still better than UPM6 and UPM7, even provided that these values were used for comparison. It should also be noted that all the geometry optimizations using UrPM6 were successful. Therefore, we anticipate that UrPM6 can be useful as a convenient and fast pre-screening tool to predict *J* values, alternative to the first-principles UDFT approach.

### 3.2. Test Set (ii): Oxidation Reactions

#### 3.2.1. Epoxidation of Ethylene

**Figure 2:**
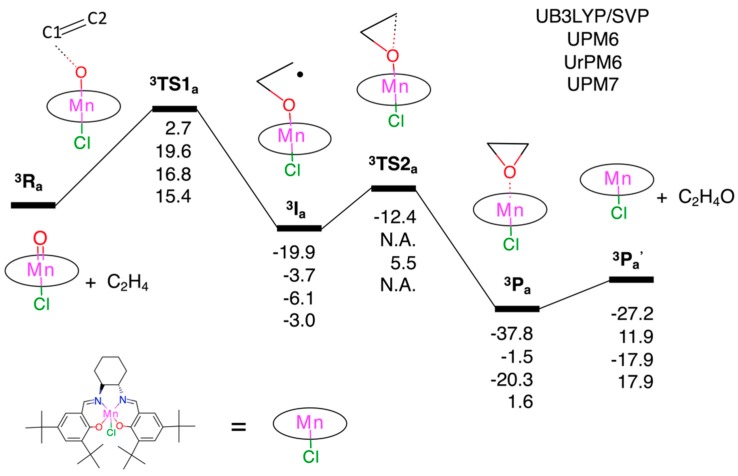
Potential energy profile with zero-point energy corrections (in kcal/mol) relative to the isolated ^3^[Cl–Mn^V^=O(salen)] complex and ethylene (**^3^R_a_**) obtained at the UB3LYP/SVP, UPM6, UrPM6, and UPM7 levels of theory. The superscript 3 on the left side denotes triplet spin state, and N.A. represents not available (Geometry optimizations failed).

As illustrated in Scheme 1a, for simplicity, the epoxidation of ethylene with ^3^[Cl–Mn^V^=O(salen)] on the triplet surface was examined in accordance with Ref. [70]. The sum of the isolated ethylene and the complex is defined as the reactant (**^3^R_a_**). The reaction starts with the electrophilic attack of the Mn^V^=O species on the π-bond of ethylene to generate an O–C1 bond via **^3^TS1_a_**. The resulting radical intermediate (**^3^I_a_**) undergoes formation of the product complex (**^3^P_a_**) involving a ring-closure barrier (**^3^TS2_a_**). Figure 2 shows the potential energy profiles (including zero-point energy corrections) for the epoxidation reaction computed at the UB3LYP/SVP, UPM6, UrPM6, and UPM7 levels of theory. 

**Figure 3:**
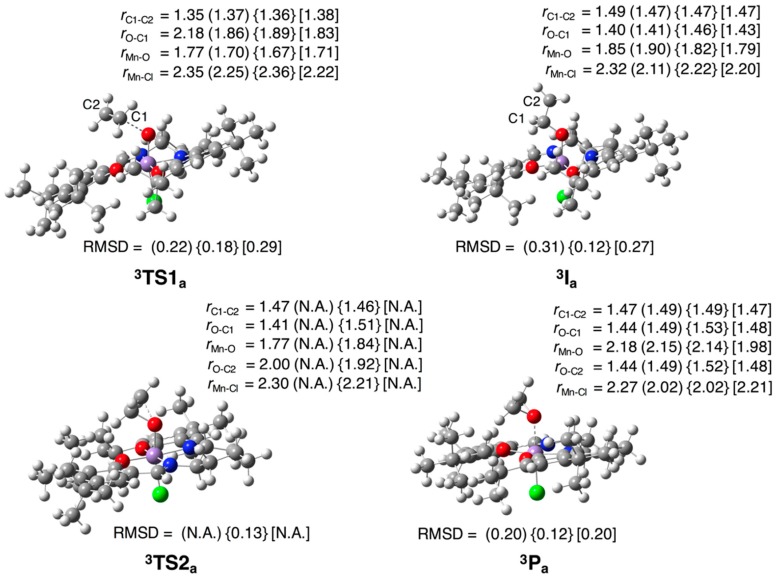
Selected optimized structural parameters of critical points in the epoxidation reaction of ethylene catalyzed by ^3^[Cl–Mn^V^=O(salen)], with bond lengths (in Å) obtained at the UB3LYP/SVP, (UPM6), {UrPM6}, and [UPM7] levels of theory, and with heavy-atom root-mean-squared deviation (RMSD) values from the reference UB3LYP-optimized structures. N.A. represents not available (The optimization failed to converge).

The rate-limiting step **^3^TS1_a_** was calculated to lie 2.7 kcal/mol above **^3^R_a_** at the UB3LYP/SVP level, and can be viewed as an early TS with a long O–C1 distance of 2.18 Å as depicted in Figure 3. The results of all SQM methods were similar to each other but significantly different from that of UB3LYP. The barrier heights were 15.4, 16.8, and 19.6 kcal/mol computed by UPM7, UrPM6, and UPM6, respectively, all of which considerably deviate from the reference UB3LYP value. The O–C1 separations at **^3^TS1_a_**, namely 1.83, 1.89, and 1.86 Å, indicate a late TS, but they did not decrease with increasing barrier height. The relative energies for **^3^I_a_** were also underestimated by more than 10 kcal/mol compared to the UB3LYP result. We are not surprised at the differences because previous statistical evaluations revealed that errors in barrier heights and reaction energies with SQM methods generally exceed 10 kcal/mol for organic molecules [46,48]. In addition, the difference in electronic structures of the ^3^[Mn^V^=O] and ^3^[Mn^IV^(O)(ethylene•)] moieties at **^3^TS1_a_** and **^3^I_a_** can also be related to the deviations. 

At **^3^TS1_a_**, the UB3LYP method favors an electronic configuration ^3^[Mn(↑↑↑)O(↓)], where an *α*-electron is transferred from ethylene to generate an intermediate ^3^[Mn(↑↑↑)(O)(ethylene•↓)] corresponding to **^3^I_a_**(see also computed spin densities listed in Table S3). The UPM6 calculation yielded a similar configuration for **^3^TS1_a_**, but ended up being ^3^[Mn(↑)(O)(ethylene•↑)] at **^3^I_a_**. In the UrPM6 and UPM7 cases, the ^3^[Mn(↑)O(↑)] configuration is favorable over ^3^[Mn(↑↑↑)O(↓)], and the former species yields another configuration for **^3^I_a_**, i.e., ^3^[Mn(↑)(O)(ethylene•↑)], by abstracting a *β*-electron from ethylene. Leaving aside quantitative inaccuracies, additional vibrational frequency calculations at the UB3LYP/SVP level ensure that the geometries optimized by the SQM methods tested here are acceptable as an initial guess for a successive refinement by UDFT. Specifically, we confirmed that an optimized geometry has only one frequency (or one large imaginary frequency, followed by much smaller one(s)), and the corresponding motion indicates the correct direction. The UrPM6-optimized stationary points can serve as the best initial guesses, because of the smallest heavy-atom root-mean-squared deviation (RMSD) values from the UB3LYP-optimized structures for **^3^TS1_a_** (0.18 Å) and **^3^I_a_** (0.12 Å).

After formation of **^3^I_a_**, the reaction proceeds smoothly. The second TS for the ring-closure (**^3^TS2_a_**) that leads to **^3^P_a_** is 7.5 kcal/mol higher in energy than **^3^I_a_**, whereas the state is more stable relative to **^3^R_a_**. The overall reaction is found to be exothermic by 37.8 kcal/mol at the UB3LYP/SVP level. For the latter stage, the SQM calculations show different tendencies. All attempts to locate **^3^TS2_a_** failed using UPM6 and UPM7. Considering the stability of ethylene oxide, both the UPM6 and UPM7 methods indicate that the relative energies at **^3^P_a_** are slightly higher than **^3^I_a_** by 2.3 and 4.6 kcal/mol, respectively. Besides, **^3^P_a_** obtained with UPM7 is found to lie above even **^3^R_a_**, which shows that the epoxidation is an endothermic process. In sharp contrast to UPM6 and UPM7, our UrPM6 is able to describe the overall reaction qualitatively, though the calculated relative energies for **^3^TS2_a_** and **^3^P_a_** are higher by 17.9 and 17.5 kcal/mol, respectively, than the UB3LYP data. A possible reason for the large differences is that the change of the electronic configuration from ^3^[Mn(↑)(O)(ethylene•↑)] to an unfavorable ^3^[Mn(↑↑↑)(O)(ethylene•↓)] should occur at **^3^TS2_a_** to form **^3^P_a_**. Another reason is that UrPM6 appears not to stabilize **^3^P_a_**, where Mn–O bond length is somewhat elongated (2.14 Å). To confirm our assumption, additional energy minimizations were performed for the isolated ^3^[Cl–Mn(III)(salen)] complex and ethylene oxide, called **^3^P_a_′**. Then, the sum of each minimum energy was calculated. The difference between UrPM6 and UB3LYP values is decreased to 9.3 kcal/mol as expected, but the error is still large. Interestingly, the energies for **^3^P_a_′** obtained with UPM6 and UPM7 are 11.9 and 17.9 kcal/mol higher than **^3^R_a_**, respectively, reconfirming these conventional SQM methods are incapable of stabilizing ethylene oxide.

#### 3.2.2. Fluorination of Toluene 

**Figure 4:**
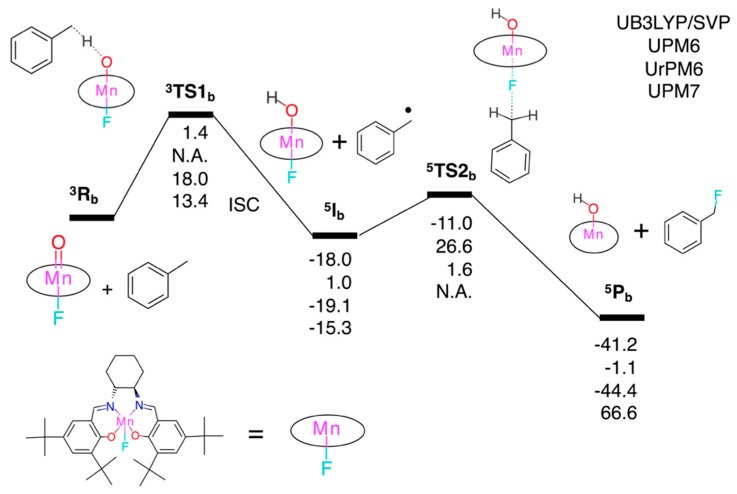
Potential energy profile with zero-point energy corrections (in kcal/mol) relative to the isolated ^3^[F–Mn^V^=O(salen)] complex and toluene (**^3^R_b_**) obtained at the UB3LYP/SVP, UPM6, UrPM6, and UPM7 levels of theory. The superscripts 3 and 5 on the left side denote triplet and quintet spin states, N.A. represents not available (Geometry optimization failed), and ISC is the acronym of intersystem crossing.

Next, let us turn to the discussion about the fluorination reaction of toluene with ^3^[F–Mn^V^=O(salen)]. According to Groves and co-workers, a high-valent Mn^V^=O is the key active species in the fluorination process, and the reaction is initiated by a hydrogen abstraction from the benzyl position of toluene via **^3^TS1_b_** that leads to an intermediate ^5^[F–Mn^IV^(OH)(salen)(benzyl radical•)] (**^5^I_b_**) involving intersystem crossing (see Scheme 1b). As in the ethylene epoxidation case discussed above, the sum of the isolated toluene and the complex is defined as the reactant (**^3^R_b_**). Subsequently, F atom transfer occurs directly from the ^5^[F–Mn^IV^(OH)] complex to the benzyl radical via **^5^TS2_b_** to form the ^5^[F–Mn^III^(salen)] complex and benzyl fluoride (**^5^P_b_**). Figure 4 displays the potential energy profiles (including zero-point energy corrections) for the fluorination reaction computed at the UB3LYP/SVP, UPM6, UrPM6, and UPM7 levels of theory. 

**Figure 5:**
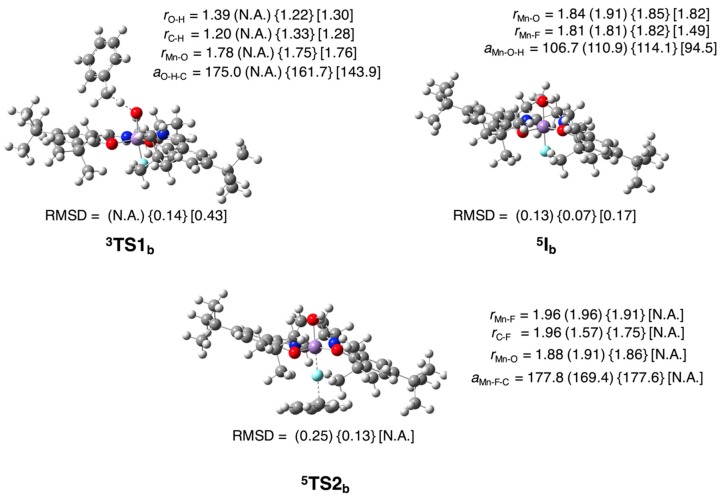
Selected optimized structural parameters of critical points in the epoxidation reaction of ethylene catalyzed by ^3^[F–Mn^V^=O(salen)], with bond lengths (in Å) and angles (in deg.) obtained at the UB3LYP/SVP, UPM6, UrPM6, and UPM7 levels of theory, and with heavy-atom root-mean-squared deviation (RMSD) values from the reference UB3LYP-optimized structures. Only ^5^[F–Mn^IV^(OH)(salen)] is shown for **^5^I_b_**. N.A. represents not available (The optimization failed to converge).

The first TS for hydrogen abstraction from the substrate (**^3^TS1_b_**) is just 1.4 kcal/mol uphill from **^3^R_b_** computed at the UB3LYP/SVP level. This is probably because of the unstable active species ^3^[F–Mn^V^=O(salen)] and weak C–H bonds of the benzylic position [78]. The optimized C–H and O–H distances of 1.20 and 1.39 Å, respectively, suggest an early TS, as can be seen in Figure 5. As we indicated in a previous work [51], attempts to find **^3^TS1_b_** failed. The latest UPM7 as well as our UrPM6 can avoid such failure, but they characterize both the electronic and geometric structures of **^3^TS1_b_** differently from the reference data obtained at the UB3LYP/SVP level. The barrier heights of 18.0 and 13.4 kcal/mol computed by UrPM6 and UPM7, respectively, significantly overestimate that by UB3LYP (1.4 kcal/mol), again due to the nature of SQM methods. Speaking of the reproducibility in geometry, the UrPM6-optimized TS structure is close to the UB3LYP-optimized one with a small RMSD value of 0.14 Å, whereas the local structure of the reaction site does not match well. The C–H and O–H distances obtained with UrPM6 (1.33 and 1.22 Å) indicate a late TS in contrast to the UB3LYP result. The UPM7 method appears to give a slightly better description, but the C–H–O angle of 143.9° is smaller than the value from UrPM6 (161.7°) and is far from the linear structure, leading to a larger RMSD value of 0.43 Å. 

Again, the difference in electronic configurations can give rise to differences in geometry and energy in the fluorination reaction studied. The electronic configuration of **^3^TS1_b_** was calculated by UB3LYP is ^3^[Mn(↑↑↑)O(↓)], whereby an *α*-electron is transferred from toluene to generate a transient intermediate ^3^[Mn(↑↑↑)(OH)(benzyl radical•↓)] that is then changed to an energetically favorable ^5^[Mn(↑↑↑)(OH)(benzyl radical•↑)] (**^5^I_b_**) via intersystem crossing (see also computed spin densities listed in Table S3). In contrast, the ^3^[Mn(↑)O(↑)] configuration is preferred to ^3^[Mn(↑↑↑)O(↓)] when using UrPM6 and UPM7. During the hydrogen abstraction, a *β*-electron of toluene is transferred to generate an intermediate ^3^[Mn(↑)(OH)(benzyl radical•↑)] that eventually turns to a quintet state identical to ^5^[Mn(↑↑↑)(OH)(benzyl radical•↑)]. The energies of **^5^I_b_** relative to **^3^R_b_** calculated by UrPM6 (−19.1 kcal/mol) and UPM7 (−15.3 kcal/mol) are close to that by UB3LYP (−18.0 kcal/mol) owing to their analogous electronic structures and the similarity in geometry. The exception, as already noted in Section 3.1, is that UPM7 favors a substantially short Mn–F bond length (1.49 Å) at **^5^I_b_**, while values from the other methods are in the range 1.81–1.82 Å. When using UPM6, an agreement with the UB3LYP result is not satisfactory. **^5^I_b_** is unlikely to be stabilized (1.0 kcal/mol above **^3^R_b_**), despite a small RMSD value of 0.13 Å, and the spin density distributions are presented in Table S3.

The UB3LYP calculation shows that the subsequent CH_2_–F bond formation via **^5^TS2_b_** requires a barrier of 7.0 kcal/mol, which is 11.0 kcal/mol below **^3^R_b_**. The rebound process provides the fluorinated product **^5^P_b_** with a large reaction energy of −41.2 kcal/mol. A linear Mn–F–C angle of 177.8° at **^5^TS2_b_** derives from the interaction between the σ* (d_z_^2^) orbital of Mn^IV^–F and the singly occupied molecular orbital of the benzyl radical [10]. Both the Mn–F and F–C separations are calculated to be 1.96 Å by means of UB3LYP. UPM6 succeeded in finding **^5^TS2_b_**, whereas the corresponding TS could not be located using UPM7. The trend is somehow the opposite to the case of **^3^TS1_b_**, and in addition, it should be strongly emphasized that neither UPM6 nor UPM7 can apply to the whole reaction. At **^5^TS2_b_**, the UPM6-optimized F–C bond length (1.57 Å) is much shorter than the Mn–F separation of 1.96 Å, and the RMSD value is calculated to be 0.25 Å. The inferior performance on the description of **^5^TS2_b_**, especially for the balance between the Mn–F and C–F bonds, causes the underestimation of the stability of **^5^P_b_**. The computed energies for **^5^TS2_b_** and **^5^P_b_** are 26.6 and −1.1 kcal/mol, respectively, relative to **^3^R_b_**. The deviation is more prominent in the case of UPM7. The crucial drawback of UPM7 is that it strongly prefers the Mn–F interaction, leading to a significant destabilization of **^5^P_b_**. The computed relative energy for **^5^P_b_** differs with the reference UB3LYP value by 107.8 kcal/mol. Our UrPM6 approach does not suffer from the inaccuracy of the conventional UPM6 and UPM7 that inherently underestimates the stability of oxidized products. Considering the characterization of **^5^TS2_b_**, the error is obviously large with a barrier of 20.7 kcal/mol, despite the better Mn–F and F–C distances of 1.91 and 1.75 Å, respectively, and a small RMSD value of 0.13 Å. In contrast, the reaction energy calculated by UrPM6 (−44.4 kcal/mol) is in good agreement with that by UB3LYP (−41.2 kcal/mol). 

Overall, we see that our UrPM6 method provides the most accurate results for the epoxidation and fluorination reactions examined here. As regards the TS searches, we had a 100% success rate from our UrPM6 and 50% from the conventional UPM6 and UPM7 methods. The UrPM6-optimized stationary points also agree well with the reference UB3LYP-optimized geometries, with an average RMSD value of 0.13 Å. Thus, these geometries can, of course, be employed as an initial guess for the following refinements by UDFT without requiring additional changes in geometric parameters. The relative energies for local minima are reproduced well. The estimation of barrier heights is a difficult issue for UrPM6, as the deviations from the reference UB3LYP results were exceeded more than 10 kcal/mol. Nevertheless, it cannot be ruled out that the B3LYP function inherently underestimates barrier heights both for closed-shell [79,80] and open-shell [81] systems.

## 4. Conclusions

As a continuation of our recent work, we presented the reparameterization of the PM6 parameter sets for fluorine and chlorine, both of which can play an important role in catalytic reactions. To avoid the degradation of the reparameterization’s accuracy for general chemical problems, several subsets in the GMTKN30 database were adopted in our training set. Then, we carried out two types of assessments. First, the UrPM6 method was applied to 25 dinuclear metal complexes to compute their *J* values. Comparison between UrPM6 and the conventional methods (UPM6 and UPM7) clearly demonstrate the superior performance of UrPM6. The MAE value obtained by UrPM6 was just 19 cm^−1^. The original UPM6 method cannot provide local minima of diiron complexes properly, and thereby strongly overestimates the experimental values by at most two digits. The latest UPM7 method performs better than UPM6 with respect to most of the dinuclear complexes tested, except for fluoride-bridged systems. Consequently, the MAEs are much higher at the UPM6 (373 cm^−1^) and UPM7 (119 cm^−1^) levels. Second, barrier heights and reaction energies for epoxidation and fluorination reactions catalyzed by manganese-oxo species were evaluated. Our UrPM6 method succeeded in locating all of the TS structures with a 100% success rate, while the success rates obtained by UPM6 and UPM7 were only 50%. The UrPM6-optimized stationary points were close to the reference UB3LYP-optimized geometries, as the maximum RMSD value was 0.18 Å. The conventional UPM6 and UPM7 inherently underestimates the stability of oxidized products, whereas the UrPM6 method can circumvent such problems. Therefore, the accuracy for estimating reaction energies is also greatly improved. The findings of this study suggest that UrPM6 can serve as an easy-to-handle and fast pre-screening method to predict *J* values, and that it has high usability for pre-optimizations of stationary points. 

## Figures and Tables

**Table 1:** Comparison of parameters ^a^ between rPM6 and PM6 ^b^.

	rPM6		PM6	
*α*_F-H_	3.293972		3.136740	
*x*_F-H_	0.851422		0.815802	
*α*_F-C_	2.984681		3.027600	
*x*_F-C_	0.736305		0.732968	
*α*_F-N_	2.899491		2.856646	
*x*_F-N_	0.567813		0.635854	
*α*_F-O_	3.051565		3.015444	
*x*_F-O_	0.694847		0.674251	
*α*_F-Mn_	2.920150		2.799150	
*x*_F-Mn_	1.077996		1.113070	
*α*_F-Fe_	4.261457		4.294707	
*x*_F-Fe_	3.657350		3.657350	
*α*_Cl-H_	2.575944		2.402886	
*x*_Cl-H_	0.946833		0.754831	
*α*_Cl-C_	2.208931		2.162197	
*x*_Cl-C_	0.550541		0.515787	
*α*_Cl-N_	2.198862		2.172134	
*x*_Cl-N_	0.586460		0.520745	
*α*_Cl-O_	2.379828		2.323236	
*x*_Cl-O_	0.642703		0.585510	
*α*_Cl-Mn_	1.951202		1.657010	
*x*_Cl-Mn_	0.162889		0.201850	
*α*_Cl-Fe_	1.574455		1.229793	
*x*_Cl-Fe_	0.004971		0.019473	
	F	Cl	F	Cl
*U_ss_*	−137.738801	−63.941830	−140.225636	−61.389930
*U_pp_*	−98.295972	−54.599897	−98.778044	−54.482801
*U_dd_*		−33.686857		−38.258155
*β_s_*	−71.770655	−4.283409	−69.922593	−2.367988
*β_p_*	−29.432906	−12.99524	−30.448165	−13.802139
*β_d_*		−1.426944		−4.037751
*ζ_s_*	5.975874	2.645837	6.043849	2.637050
*ζ_p_*	2.932020	2.084940	2.906722	2.118146
*ζ_d_*		1.291613		1.324033

^a^ In Å^−1^ for *α*, eV for *U* and *β*, and bohr^−1^ for *ζ*. ^b^ From Ref. [53].

**Table 2:** Magnetic exchange coupling constants (in cm^−1^) for 25 dinuclear metal complexes.

System		Expt.	UPM6	UrPM6	UPM7
FEBKOM	Mn^IV^_2_(μ–O)_2_	−144	−9	−130	−39
FEBKUS	Mn^III^Mn^IV^(μ–O)_2_	−148	−49	−132	−32
HUYGAJ	Mn^IV^_2_(μ–O)_2_	−45	14	−70	−30
GAVWIJ	Mn^III^Mn^IV^(μ–O)_2_	−114	−10	−110	−21
VEGTEG	Mn^II^–OH–Mn^II^	−9	−6	−6	−14
TIPFED	Mn^II^–OH–Mn^III^	−9	−0.4	−5	−17
PIZWIG	Mn^III^–F–Mn^III^	−17	−16	−12	−470
EDEWIV	Mn^II^–F–Mn^II^	−3	−354	−5	−121
CULSOR	Mn^III^–O–Mn^III^	9	−5	3	−18
ARUGOK	Mn^III^–O–Mn^III^	3	−5	7	−25
ARUGUQ	Mn^III^–O–Mn^III^	−13	−8	−35	N.A. ^1^
ILELEQ	Mn^II^Mn^II^	−4	7	−6	−5
KEJWIG	Mn^II^Mn^III^	−4	6	6	2
BARFOQ	Mn^II^Mn^III^	−1	N.A. ^1^	−27	N.A. ^1^
YOCKAC	Fe^III^_2_(μ–O)_2_	−27	−955	−95	−804
sMMOQ ^2^	Fe^IV^_2_(μ–O)_2_	−224 ^3^	−1311	−201	−27
UCANAP	Fe^III^_2_(μ–OH)_2_	−18	−1217	−33	−104
CEPBEF	Fe^III^–OH–Fe^III^	−42	−1252	−27	−91
EDEVAM	Fe^II^–F–Fe^II^	−8	−194	−0.2	−191
KASPEC	Fe^II^–Cl–Fe^II^	−17	−38	−16	−187
KEYXOC	Fe^III^–O–Fe^III^	−106	−1149	−93	−72
QEZHIN	Fe^III^–O–Fe^III^	−212	−360	−118	−98
GUQMUA	Fe^II^–O–Fe^III^	−119	−459	−114	−70
RINXUJ	Fe^III^Fe^III^	−29	−648	−51	−74
RITHAD	Fe^III^Fe^III^	−10	−1010	−98	−78
MAE ^4^			373	19	119
MARE ^5^			18.5	1.0	7.0

^1^ Geometry optimizations failed to converge. ^2^ A model complex for the active site of soluble methane monooxygenase Q (sMMOQ). ^3^ The value in parenthesis is obtained by the UB3LYP calculation taken from Ref. [77]. ^4^ Mean absolute errors (MAEs) are calculated excluding the results of BARFOQ and ARUGUQ. ^5^ Mean absolute relative errors (MAREs) are calculated excluding the results of BARFOQ and ARUGUQ.

## Supplementary Materials

The following are available online. Table S1: Compounds with charge, spin multiplicity used in training sets **1** and **2** for fluorine and chlorine, Figure S1: Illustration of the Mn and Fe complexes in Table S1, Table S2: List of names used in the test set to evaluate the performance on magnetic interactions, Figure S2: Illustration of local minima for EDEVIW optimized by UPM6, UrPM6, and UPM7, Table S3: Spin density distributions for stationary points obtained at the UB3LYP/SVP, UPM6, UrPM6, and UPM7 levels of theory.
