# Supplementary material for: Quantitative Assessment of rPM6 for Fluorine- and Chlorine-Containing Metal Complexes: Comparison with Experimental, First-Principles, and Other Semiempirical Results
Source: Molecules. 2018 Dec 15;23(12):3332. doi: 10.3390/molecules23123332 (PMC6321459; doi:10.3390/molecules23123332)
Supplement: Supplementary file 1 [file molecules-23-03332-s001.zip › supplementary.pdf]

Supplementary Materials

Quantitative Assessment of rPM6 for fluorine- and chlorine-containing metal complexes: Comparison with experimental, first-principles, and other semiempirical results

Toru Saito *, Manami Fujiwara and Yu Takano

Graduate School of Information Sciences, Hiroshima City University, 3-4-1 Ozuka-Higashi, Asa-Minami-Ku, Hiroshima 731-3194; tsaito@hiroshima-cu.ac.jp (T.S.); fujiwara@bio.info.hiroshima-cu.ac.jp (M.F.); ytakano@hiroshima-cu.ac.jp (Y.T.)

* Correspondence: tsaito@hiroshima-cu.ac.jp; Tel.: +81-82-830-1617

Table S1. Compounds with (charge, spin multiplicity), used in training sets **1** and **2** for fluorine and chlorine.

F		Cl	
CH_2F_2	(0, 1)	CH_3Cl	(0, 1)
CH_2NH_2F	(0, 1)	CH_2Cl_2	(0, 1)
CH_2OHF	(0, 1)	CH_2ClF	(0, 1)
CH_3COF	(0, 1)	CH_2NH_2Cl	(0, 1)
CHF_3	(0, 1)	CH_2OHCl	(0, 1)
FO	(0, 2)	CH_3COCl	(0, 1)
FOF	(0, 1)	$CHCl_3$	(0, 1)
FOH	(0, 1)	Cl_2O	(-1, 1)
FOO	(0, 2)	$HClO_4$	(0, 1)
FOOF	(0, 1)	HOCl	(0, 1)
NH_3F	(1, 1)	NCl_3	(0, 1)
N_2F	(1, 1)	NH_2Cl	(0, 1)
C_6H_5F	(0, 1)	$NHCl_2$	(0, 1)
Mn(IV)(Por)F_2 [a]	(0, 4)	NOCl	(0, 1)
Fe(III)(cyclamacetate) F [b]	(1, 2)	C_6H_5Cl	(0, 1)
		Mn(II)(tpa)Cl_2 [c]	(0, 6)
		Fe(IV)=O(TMC)Cl [d]	(1, 5)
		Fe(III)(Py_2TTA)Cl_2 [e]	(0, 6)

[a] See Fig. S1 (a)

[b] See Fig. S1 (b)

[c] tpa = tris-2-picolylamine, see Fig. S1 (c)

[d] TMC = 1,4,8,11-tetramethyl-1,4,8,11-tetraazacyclotetradecane

[e] Py_2TTA = 3,5-bis(2-pyridyl)-1,2,4,6-thiatriazine

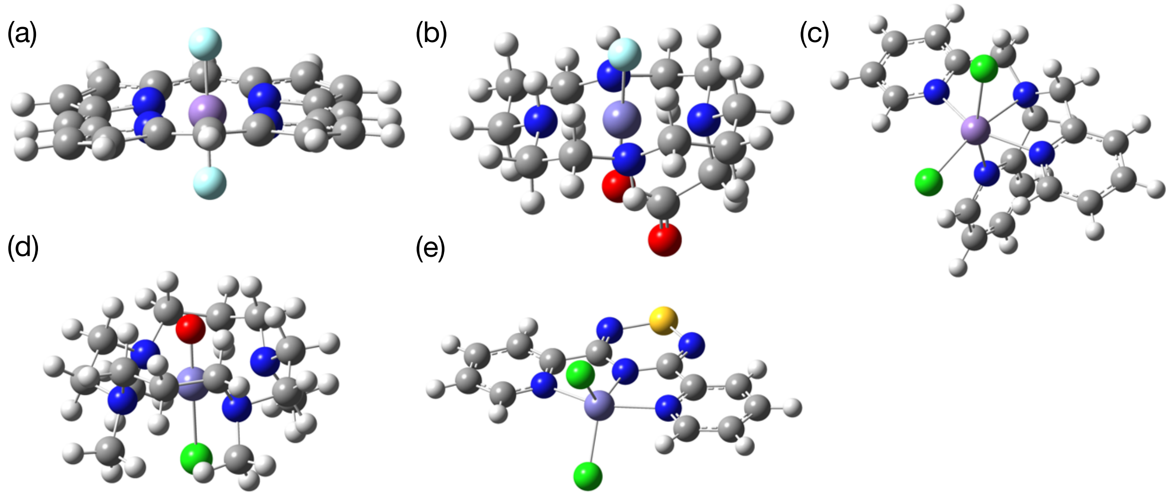

Figure S1. Illustration of the Mn and Fe complexes in Table S1.

Table S2. List of names used in the test set to evaluate the performance on magnetic interactions.

System	
FEBKOM	bis(μ2-oxo)-bis(bis(1,10-phenanthroline-*N,N'*)-Mn(IV))
FEBKUS	bis(μ2-oxo)-bis(bis(1,10-phenanthroline-*N,N'*))-Mn(III)Mn(IV)
HUYGAJ	bis((μ2-acetato)-bis(μ2-oxo)-bis(2,2'-bipyridyl)-dichloro-di-Mn(IV))
GAVWIJ	(μ2-acetato-*O,O'*)-bis(μ2-oxo)-dichloro-bis(2,2'-bipyridine-*N,N'*)-Mn(III)Mn(IV)
TIPFED	(μ2-hydroxo)-bis(μ2-pivalato-*O,O'*)-bis(1,4,7-trimethyl-1,4,7-triazacyclononane)-Mn(II)Mn(III)
VEGTEG	(μ2-hydroxo)-bis((μ2-acetato-*O,O'*)-(*N,N',N''*-trimethyl-1,4,7-triazacyclononane-*N,N',N''*)-di-Mn(II))
KEJWIG	(μ2-2,6-bis((2-(2-pyridyl)ethyl)(2-pyridylmethyl)aminomethyl)-4-methylphenolato)-bis(μ2-acetato)-Mn(II)Mn(III)
ILELEQ	bis(μ2-acetato)-(μ2-2,6-bis(bis(2-pyridylmethyl)aminomethyl)-4-nitrophenolato)-di-Mn(II)
PIZWIG	(μ2-fluoro)-tetrafluoro-bis(1,4,7-trimethyl-1,4,7-triazonane)-di-Mn(III)
EDEWIV	(μ2-fluoro)-bis(μ2-1,3-bis[bis(3,5-dimethyl-1-pyrazolyl)-methyl]benzene)-di-Mn(II)
CULSOR	(μ2-oxo)-bis(μ2-acetato-*O,O'*)-bis(1,4,7-triazacyclononane-N^1,N^4,N^7)-di-Mn(III)
ARUGOK	bis(μ2-2-chlorobenzoato)-(μ2-oxo)-bis(1,10-phenanthroline)-diaqua-di-Mn(iii)
ARUGUQ	bis(μ2-2-chlorobenzoato)-(μ2-oxo)-bis(1,10-phenanthroline)-diaqua-di-Mn(iii)
BARFOQ	bis(μ2-*N*-methyl-*N'*-carboxymethyl-*N,N'*-bis(2-pyridylmethyl)ethane-1,2-diamine)-diaqua-di-Mn(II)
KASPEC	bis(μ-(1,1'-((3-(bis(3,5-dimethyl-1H-pyrazol-1-yl)methyl)phenyl)methylene)bis(3,5-dimethyl-1H-pyrazole)))-(μ-chloro)-di-Fe(II)
EDEVAM	(μ2-fluoro)-bis(μ2-1,3-bis[bis(3,5-dimethyl-1-pyrazolyl)-methyl]benzene)-di-Fe(II))
UCANAP	bis(μ2-hydroxo)-tetrakis(bis(1-methylimidazol-2-yl) ketone)-di-Fe(III)
CEPBEF	(μ2-hydroxo)-bis(4-azaheptane-1,7-bis(salicylideneiminate))-di-Fe(III)
YOCKAC	bis((μ2-oxo)-(tris((6-methylpyrid-2-yl)methyl)amine-*N,N',N''*)-Fe(III))
RINXUJ	bis(μ2-(2-(((2-(oxy)-3-((2-(oxy)-3-methoxybenzylidene)amino)propyl)imino)methyl)-6-ethoxyphenolato))-di-Fe(III)
KEYXOC	(μ2-oxo)-difluoro-bis(tris(2-pyridylmethyl)amine)-di-Fe(III)
QEZHIN	(μ2-oxo)-bis(2,4,6-tris(2-pyridyl)-1,3,5-triazine-*N,N',N''*)-bis(dicyanamide-N)-bis(nitrato-*O,O'*)-di-Fe(III)
RITHAD	(μ2-Acetato-*O,O'*)-bis(μ2-methoxo)-bis((2-oxybenzyl)-(2-(imidazol-2-yl)ethyl)amine-*N,N',O*)-di-Fe(III)
GUQMUA	(μ2-oxo)-bis(μ2-triphenylethanolato)-bis(1,4,7-trimethyl-1,4,7-triazacyclononane)-Fe(II)Fe(III)

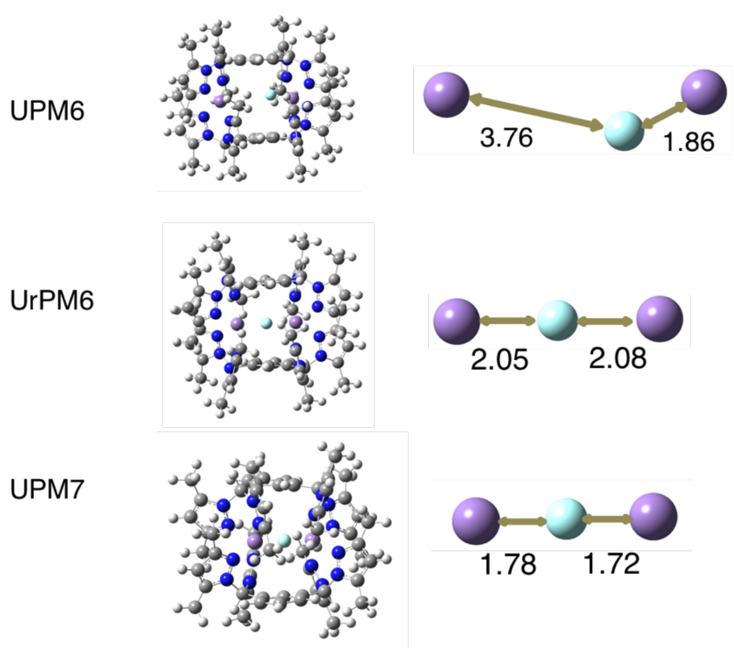

Figure S2. Illustration of local minima for EDEVIW optimized by UPM6, UrPM6 and UPM7

Table S3. Spin density distributions for stationary points obtained at the UB3LYP/SVP, UPM6, UrPM6, and UPM7 levels of theory.

Species	Method	ρ_{Mn}	ρ_{O}	ρ_{Sub}	ρ_{rest}
**^{3}TS1$_a$**	UB3LYP	2.90	–0.64	–0.23	–0.03
	UPM6	2.42	–0.25	–0.18	0.01
	UrPM6	1.06	0.79	0.21	–0.06
	UPM7	1.08	0.62	0.33	–0.03
**^{3}I$_a$**	UB3LYP	2.98	0.04	–0.99	–0.03
	UPM6	1.02	–0.05	1.01	0.02
	UrPM6	1.10	–0.13	1.02	0.01
	UPM7	0.99	–0.06	1.02	–0.05
**^{3}TS$_a$**	UB3LYP	2.48	0.12	–0.54	–0.06
	UPM6	N.A.	N.A.	N.A.	N.A.
	UrPM6	1.44	0.03	0.59	–0.06
	UPM7	N.A.	N.A.	N.A.	N.A.
**^{3}P$_a$**	UB3LYP	1.94	–0.01	0.00	0.07
	UPM6	2.95	–0.02	0.02	–0.95
	UrPM6	2.01	–0.02	0.01	0.00
	UPM7	2.02	–0.01	0.01	–0.02
**^{3}TS1$_b$**	UB3LYP	2.84	–0.54	–0.31	0.01
	UPM6	N.A.	N.A.	N.A.	N.A.
	UrPM6	1.10	0.54	0.42	–0.06
	UPM7	0.91	0.66	0.34	0.09
**^{5}I$_b$**	UB3LYP	2.93	0.04	1.00	0.03
	UPM6	3.00	–0.01	1.00	0.01
	UrPM6	3.08	0.00	1.00	–0.08
	UPM7	2.81	0.04	1.00	0.15
**^{5}TS2$_b$**	UB3LYP	3.42	0.10	0.60	–0.12
	UPM6	3.33	0.08	0.56	0.03
	UrPM6	3.32	0.11	0.68	–0.11
	UPM7	N.A.	N.A.	N.A.	N.A.

N.A.: not available.
